# Custom microarray construction and analysis for determining potential biomarkers of subchronic androgen exposure in the Eastern Mosquitofish (*Gambusia holbrooki*)

**DOI:** 10.1186/1471-2164-14-660

**Published:** 2013-09-28

**Authors:** Erica K Brockmeier, Fahong Yu, David Moraga Amador, Timothy A Bargar, Nancy D Denslow

**Affiliations:** 1 Department of Physiological Sciences, Center for Environmental and Human Toxicology, University of Florida, 2187 Mowry Road, P.O. Box 110885, 32611 Gainesville, FL, USA; 2 Interdisciplinary Center for Biotechnology Research, University of Florida, P.O. Box 103622, 32611 Gainesville, FL, USA; 3 Southeast Ecological Science Center, U.S. Geological Survey, 7920 NW 71st Street, 32653 Gainesville, FL, USA; 4 Genetics Institute, University of Florida, 2033 Mowry Road, P.O. Box 103610, 32610 Gainesville, FL, USA

**Keywords:** *Gambusia holbrooki*, 17β-trenbolone, Androgen biomarker, Aquatic toxicology, Ecotoxicogenomics, Microarray, Gonopodium

## Abstract

**Background:**

The eastern mosquitofish (*Gambusia holbrooki*) has the potential to become a bioindicator organism of endocrine disrupting chemicals (EDCs) due to its androgen-driven secondary sexual characteristics. However, the lack of molecular information on *G*. *holbrooki* hinders its use as a bioindicator coupled with biomarker data. While traditional gene-by-gene approaches provide insight for biomarker development, a holistic analysis would provide more rapid and expansive determination of potential biomarkers. The objective of this study was to develop and utilize a mosquitofish microarray to determine potential biomarkers of subchronic androgen exposure. To achieve this objective, two specific aims were developed: 1) Sequence a *G*. *holbrooki* cDNA library, and 2) Use microarray analysis to determine genes that are differentially regulated by subchronic androgen exposure in hepatic tissues of 17β-trenbolone (TB) exposed adult female *G*. *holbrooki*.

**Results:**

A normalized library of multiple organs of male and female *G*. *holbrooki* was prepared and sequenced by the Illumina GA IIx and Roche 454 XLR70. Over 30,000 genes with e-value ≤ 10^-4^ were annotated and 14,758 of these genes were selected for inclusion on the microarray. Hepatic microarray analysis of adult female *G*. *holbrooki* exposed to the vehicle control or 1 μg/L of TB (a potent anabolic androgen) revealed 229 genes upregulated and 279 downregulated by TB (one-way ANOVA, p < 0.05, FDR α = 0.05, fold change > 1.5 and < −1.5). Fifteen gene ontology biological processes were enriched by TB exposure (Fisher’s Exact Test, p < 0.05). The expression levels of *17β*-*hydroxysteroid dehydrogenase 3* and *zona pellucida glycoprotein 2* were validated by quantitative polymerase chain reaction (qPCR) (Student’s t-test, p < 0.05).

**Conclusions:**

Coupling microarray data with phenotypic changes driven by androgen exposure in mosquitofish is key for developing this organism into a bioindicator for EDCs. Future studies using this array will enhance knowledge of the biology and toxicological response of this species. This work provides a foundation of molecular knowledge and tools that can be used to delve further into understanding the biology of *G*. *holbrooki* and how this organism can be used as a bioindicator organism for endocrine disrupting pollutants in the environment.

## Background

The eastern and western mosquitofish (*Gambusia holbrooki* and *G*. *affinis* respectively) are members of the live bearer family *Poeciliidae* that are collectively the most widely distributed freshwater fish species in the world [1]. Males and females are small in size (1–2 cm total length for males and 3–5 cm for females) and exhibit sexual dimorphism. Sexually-mature male mosquitofish have an elongation of anal fin rays 3, 4, and 5; this structure is referred to as the gonopodium and is used by males for internal fertilization of females [2]. Abnormal anal fin growth which resembles the male gonopodium can be induced in female mosquitofish by exposure to androgenic compounds [2-6].

Mosquitofish attracted the attention of environmental toxicologists after the discovery of abnormally elongated anal fins on female *G*. *holbrooki* residing downstream of a pulp and paper mill in Florida [7]. It was hypothesized that these fish were exposed to an androgenic chemical since male sex steroids induce similar phenotypes [2]. Studies utilizing the mosquitofish as a bioindicator for evaluating the impacts of paper mill effluent exposure have provided useful information on the evaluation of the physiological impacts of this exposure [8,9]; however, the identification of the chemical or class of chemicals inducing the abnormal elongation could not be elucidated from these studies.

The development and usage of gene expression biomarkers can provide knowledge on the effects at a molecular level that chemicals have on an organism [10]. Traditionally, biomarkers have been found with a gene-by-gene approach by utilizing knowledge on mechanisms of action to determine potential biomarker genes. A classic example in the field of ecotoxicology is hepatic expression of the egg yolk precursor protein *vitellogenin* (*vtg*), a gene normally only expressed in female fish but whose expression in males can be driven by exogenous exposure to estrogenic compounds [11]. For mosquitofish, potential biomarkers of androgen exposure that are expressed in the androgen-sensitive anal fin tissue include *sonic hedgehog* (*shh*), *fibroblast growth factor receptor 1* (*fgfr1*), and *muscle segment homeobox C* (*msxC*) [4,12]. While this gene-by-gene approach has been useful in the past, a method that includes the analysis of many genes at a single time could generate larger data sets for more quickly developing tools to evaluate the impacts of chemical stressors in the environment.

Microarray technologies provide a way of evaluating the expression of thousands of genes in a single sample. This platform has enabled the field of ecotoxicology to develop chemical gene expression signatures, to better understand the mechanisms of action of chemical exposure, and to elucidate potential biomarker genes [13,14]. While there are commercial microarrays for several model organisms such as zebrafish (*Danio rerio*), medaka (*Oryzias latipes*), and the fathead minnow (*Pimephales promelas*) and for commercially-important species including the Atlantic salmon (*Salmo salar*) and largemouth bass (*Micropterus salmoides*) [14], there is currently no microarray for *G*. *holbrooki* or *G*. *affinis*. This type of molecular tool could be used to further develop the mosquitofish as a robust bioindicator organism for endocrine disrupting chemicals (EDCs) by providing a better understanding of the molecular mechanisms of chemical exposure on this species and can provide a means to elucidate potential androgen biomarker genes.

The objective of this study was to develop and utilize a mosquitofish microarray to determine potential biomarkers of subchronic androgen exposure. To achieve this objective, two specific aims were developed: 1) Sequence a *G*. *holbrooki* cDNA library, and 2) Use microarray analysis to determine genes that are differentially regulated by subchronic androgen exposure in hepatic tissues of 17β-trenbolone (TB) exposed adult female *G*. *holbrooki*. By using the custom *G*. *holbrooki* microarray, we identified a set of genes that were significantly differentially regulated after 14 days of TB exposure and hypothesize that these genes could be used as androgenic biomarkers.

## Results

### *G*. *holbrooki* cDNA library sequencing and microarray construction

Table 1 depicts the results of the two sequencing analyses that were conducted. Over 700,000 contigs were obtained from the combined 454 XLR70 GS-FLX and Illumina GAIIx sequencing runs, with a greater number of large contigs obtained using the 454 XLR70 GS-FLX. In total, 285,780 sequences were obtained from both the 454 and Illumina runs after co-assembly of the two data sets using PTA, with 165,062 final assemblies passing quality control checks and used for annotation analysis. After annotation, 31,160 sequences were found which had an e-score of ≤ 10^-4^, indicating that these sequences were well-annotated and appropriate for inclusion in the microarray.

**Table 1:** ***G. holbrooki *cDNA library sequencing and assembly results**

**Roche 454 sequencing**		**Illumina sequencing**	
Total number of clean bases	1,338,668,195	Total number of bases	67,321,337
Number of fully assembled reads	8,283,907	Number of clean reads	13,930,835
Number of contigs	162,704	Number of contigs	568,658
Average contig size	256.95	Average contig size	133.25
Range of contig length	100 – 3,413	Range of contig length	50 – 2,976
*454 and Illumina co-assembly results*			
Number of starting input sequences		285,780	
Number of sequences kept after clean-up		213,296	
Number of final assemblies		165,062	
Number of gene hits with e-value ≤ 10^-4^		31,160	
Number of sequences with matches to *Homo sapiens*		27,505	
Number of sequences with matches to *Danio rerio*		31,420	

The gene selection for microarray design was based on the strength of gene annotation. To find a maximum set of non-redundant sequences representing unique genes, we selected a subset of genes for probe design from the BLAST results with e-values of  ≤ e^-7^ to ≤ e^-10^ as the cut-off thresholds in the NR & NT search and RefSeq search respectively. Selected sequences were chosen in order to obtain an overall breadth of diverse biological and molecular pathways. 60-mer gene probes were designed for 14,758 genes for the microarray using eArray (Agilent, Santa Clara, USA). The microarray platform (GPL16784) and resulting data set (GSE45261) were submitted to the Gene Expression Omnibus (GEO) database. All reporting and depositing of microarray information was conducted per the guidelines of the Minimum Information About a Microarray Experiment (MIAME) [15].

### Microarray analysis of hepatic gene expression patterns

**Figure 1:**
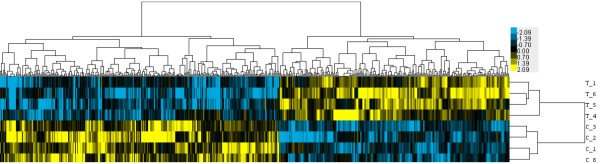
**Gene expression differences between the control and 17β-trenbolone (TB) exposed female *G*. *holbrooki *as determined by hierarchical cluster analysis.** Genes with fold changes > 1.5 fold or < −1.5 fold over controls and with one-way ANOVA p-values < 0.05 were used for this analysis, and 279 genes were found to be down-regulated by TB exposure and 229 genes up-regulated by TB exposure. Data were median-centered by gene and clustered using centered correlation and complete linkage. Yellow genes are more highly expressed than the gene average and blue genes are expressed at a lower level than the gene average. The resulting gene expression map demonstrates the distinct hepatic expression profiles of the vehicle control-exposed and TB exposed groups.

Figure 1 depicts the differentially-regulated transcripts by 1 μg TB/L exposure with a greater than 1.5 fold increase or a less than −1.5 fold decrease from the controls which were significantly different between treatments (one-way analysis of variance (ANOVA), p < 0.05, false discovery rate α = 0.05). Distinct hepatic gene expression profiles between the two treatment groups were visualized using hierarchical cluster analysis (Figure 1). Under these conditions, 279 genes were down-regulated by TB as compared to the vehicle control and 229 genes were up-regulated by exposure to TB. A list of all significant differentially-regulated transcripts with a greater than 1.5 fold increase or a less than −1.5 fold decrease from the controls can be found in the supplemental materials (Additional file 1).

Table 2 demonstrates the gene ontology (GO) biological process categories that were significantly enriched by TB exposure as determined by gene set enrichment analysis. The percent of transcripts differentially regulated (‘dif. reg’) versus those which were not differentially regulated (‘not dif. reg)’ associated with each GO category are also included in the table. These results demonstrate the percentage of genes within a GO category which were significantly differentially regulated (as indicated by the p-value resulting from the one-way ANOVA) versus genes within the GO category that were on the array which were not significant. Fifteen biological processes were enriched by TB exposure (Fisher’s Exact test, p < 0.05). Many of these processes are related to metabolism and metabolic processes (e.g. cholesterol transport and efflux) and other molecular-level impacts (e.g. positive regulation of translation) are also present.

**Table 2:** Significantly differentially regulated biological processes during TB treatment as determined by gene set enrichment analysis

**Gene Ontology Biological Process**	**Fisher’s Raw p-value**	**Type of regulation**	**Dif. reg**^**a**^	**Not dif. reg**^**b**^
go:0006629; lipid metabolic process	0.002	Enriched	1.36%	0.72%
go:0008380; rna splicing	0.047	Enriched	1.18%	0.76%
go:0015986; atp synthesis coupled proton transport	0.001	Enriched	0.73%	0.26%
go:0051246; regulation of protein metabolic process	0.018	Enriched	0.47%	0.17%
go:0051028; mrna transport	0.025	Enriched	0.37%	0.13%
go:0030168; platelet activation	0.046	Enriched	0.36%	0.15%
go:0032313; regulation of rab gtpase activity	0.046	Enriched	0.36%	0.15%
go:0042157; lipoprotein metabolic process	0.0001	Enriched	0.35%	0.02%
go:0006695; cholesterol biosynthetic process	0.0005	Enriched	0.30%	0.02%
go:0030301; cholesterol transport	0.013	Enriched	0.24%	0.04%
go:0008203; cholesterol metabolic process	0.004	Enriched	0.18%	0%
go:0033344; cholesterol efflux	0.022	Enriched	0.18%	0.02%
go:0045727; positive regulation of translation	0.040	Enriched	0.16%	0.02%
go:0006044; n-acetylglucosamine metabolic process	0.032	Enriched	0.12%	0%
go:0046677; response to antibiotics	0.032	Enriched	0.12%	0%

a. The percent of transcripts in the GO category which were differentially regulated.

b. The percent of transcripts in the GO category which were not differentially regulated.

**Figure 2:**
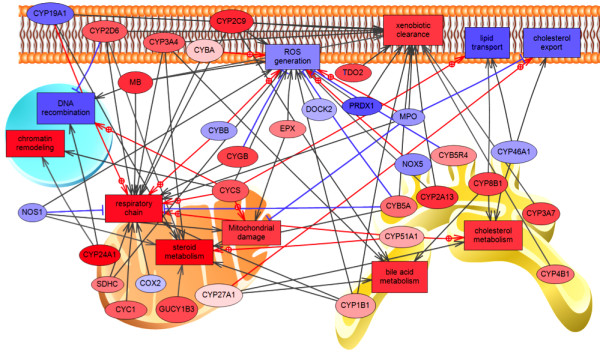
**Pathway analysis of hepatic genes differentially regulated by 17β-trenbolone: Impacts on metabolic pathways.** Connections between significantly up or down-regulated genes in the liver and enriched Biological Processes from human homolog data were visualized. The red color represents up-regulation by TB and blue color represents down-regulation by TB compared to the samples exposed to the vehicle control. The intensity of the color is correlated with the degree of differential regulation. Red solid lines represent positive regulation of gene expression, blue solid lines represent negative regulation of gene expression, and grey solid lines represent unknown direction of regulation. Oval shapes represent individual genes and boxes represent GO Biological Processes. Gene abbreviations: COX2: cyclooxygenase II; CYBA: cytochrome b-245, alpha polypeptide; CYBB: cytochrome b-245, beta polypeptide; CYB5A: cytochrome b5 type A (microsomal); CYC1: cytochrome c-1;CYCS: cytochrome c, somatic; CYGB: cytoglobin; CYP(number)(letter)(number) for all entries: cytochrome P450, family number, subfamily letter, polypeptide variant number; DOCK2: dedicator of cytokinesis 2; EPX: eosinophil peroxidase; GUCY1B3: guanylate cyclase 1, soluble, beta 3;MB: myoglobin; MPO: myeloperoxidase; NOS1: nitric oxide synthase 1; NOX5: NADPH oxidase, EF-hand calcium binding domain 5; PRDX1: peroxiredoxin 1; SDHC: succinate dehydrogenase complex, subunit C, integral membrane protein, 15 kDa; TDO2: tryptophan 2,3-dioxygenase.

Figure 2 illustrates the impacts of TB exposure on processes linked to metabolism and biosynthesis using PathwayStudio^TM^, providing further support for the results of the Fisher’s exact test. A significant increase in the processes of cholesterol metabolism, steroid metabolism, and respiratory chain are correlated with an increase in the expression of genes linked to these processes, including many cytochrome P450 enzyme subtypes (Figure 2).

### Real-time quantitative PCR (qPCR) validation of selected genes

Table 3 provides the results of the qPCR validation of the expression levels of a subset of genes selected from the microarray data. From the list of significantly up and down-regulated transcripts, five genes were selected for follow-up analysis by qPCR based on the magnitude of change in expression level, their inclusion in a significantly differentially regulated biological process, or relationship to the endocrine system: *17β*-*hydroxysteroid dehydrogenase 3* (*17βhsd3*), *androgen receptor beta* (*ARβ*), *zona pellucida glycoprotein 2* (*zp2*), *activating transcription factor 1* (*atf1*) and *acetyl*-*CoA acyltransferase 2* (*acaa2*). The ribosomal protein L8 (*rpl8*) was used for normalization of expression values and data are presented as log2-transformed fold change values. Two genes were found to be significantly expressed by qPCR in the same direction of expressional change as in the microarray data set: *17βhsd3* and *zp2* (Student’s t-test, p < 0.05). Three genes, *Atf1*, *ARβ*, and *acaa2* were also expressed in the same direction as the microarray samples, albeit the qPCR results were not statistically significant (Student’s t-test, p > 0.05).

**Table 3:** Follow-up qPCR of gene expression changes on selected genes

**Gene name**	***Array (N = 4)***	**p-value**	***qPCR (N = 8)***	**p-value**
	**Log2 change over control**		**Log2 change over control**	
*17*-*beta hydroxysteroid dehydrogenase 3*	3.16	0.019	1.831	0.028
*Activating transcription factor 1*	3.05	0.032	1.860	0.188
*Acetyl*-*CoA acyltransferase 2*	2.40	0.031	0.951	0.405
*Androgen receptor beta*	−1.32	0.033	−0.486	0.505
*zona pellucida glycoprotein 2*	−6.487	0.0001	−7.6603	<0.001

## Discussion

Transcriptomic analyses provide a promising means of furthering the field of environmental toxicology, with several applications having been realized in full and other applications, including biomarker development, having proof-of-concept models in place [14]. As researchers continue to branch out into the study of more complex systems and environments, a need for developing molecular tools for environmentally-relevant and non-model species has become evident. In this manuscript we describe the development and use of a custom gene microarray for *G*. *holbrooki*, a potential bioindicator organism for EDCs. Through the sequencing of a *G*. *holbrooki* library, we were able to generate gene sequence information for over 30,000 genes and have developed an 8 x 15,000 gene microarray.

Controlled exposures to androgenic compounds provide insightful data on the toxic mechanism of exposures to environmental androgens. This is especially true for mosquitofish, a species with androgen-driven sexual dimorphism. This is significant for environmental health since elongation of the anal fin only found in males is present in populations of female mosquitofish residing downstream of pulp and paper mills with effluents known to contain EDCs [7,16-18]. To better understand the impacts of androgen exposure on *G*. *holbrooki* and to determine potential molecular biomarkers of androgen exposure, we analysed gene expression patterns in female *G*. *holbrooki* that were exposed for 14 days to 1 μg/L of the potent model androgen TB. A dose and time point was selected that resulted in significant anal fin elongation over the controls to provide a physiological anchor of androgenic effects for these microarray data [12]. Furthermore, because of our interest in discovering biomarkers that would be expressed during long-term exposures (i.e. in organisms that reside at a contaminated site), we chose to conduct a microarray analysis of *G*. *holbrooki* that had been exposed for two weeks *in lieu* of an acute exposure.

Previous transcriptomic analysis of TB exposures on the toxicological model species, the fathead minnow (*Pimephales promelas*), have provided insights into the mechanisms of the endocrine disrupting effects caused by this chemical. Analysis of gonadal gene expression profiles in female fathead minnows exposed to 1 μg TB/L for 4 days resulted in a larger number of downregulated GO biological processes. Many of these processes are similar to those seen up-regulated in the liver of *G*. *holbrooki* by a longer term exposure to the same dose of TB (Table 2). Notably, processes related to lipid metabolism and transcription are regulated in different directions [19]. This may be due to differences in adaptation between short and longer term exposures, as different compensatory pathways may become activated at early time points and decreased at later time points to ameliorate the biological effects of the exposure [20]. This may also be due to differences in the tissue analyzed (gonad versus liver). In hepatic gene expression analysis of female fathead minnows exposed to 0.5 μg TB/L for 48 hours, there are strong similarities to the enriched GO biological processes that were found in the data set presented in this manuscript. Notably, both data sets contain pathways related to lipid metabolism and catabolism, as well as steroid metabolism and biosynthesis (Garcia-Reyero, manuscript in preparation).

Many of the enriched significant pathways in the mosquitofish TB data were associated with lipid and cholesterol metabolism and were driven by the increased expression of genes such as *apolipoprotein A1* (*apoA*-*1*), *phosphatidylcholine*-*sterol acyltransferase*, and *lecithin*-*cholesterol acyltransferase* (Additional file 1). Similar results were demonstrated in castrated rats which were fed 1 μg/g of testosterone for 14 days, with increased hepatic synthesis of *apoA*-*1*[21]. As the cholesterogenic pathway is important for its role in producing the precursor chemicals for steroid biosynthesis, the finding that androgen exposure can upregulate pathways involved in cholesterol and lipid metabolism is of interest. Even though these two chemicals differ in their ability to become aromatized, with testosterone metabolized to estrogenic forms whereas TB in contrast is non-aromatizable and instead exhibits anti-estrogenic effects [22], the similarities seen in the impacts at the pathway level indicate that androgens elicit their effects via general disruption of metabolism. These include modulations in the GO Biological Processes of lipid metabolic process, cholesterol biosynthetic process, and regulation of protein metabolic process (Table 2).

Significant additional impacts at the gene level can be visualized using PathwayStudio™ and demonstrate the impacts of TB on cytochrome P450 enzymes. Other studies have also shown an increase in certain subtypes of P450s by TB exposure [19] and the increased expression of these key metabolic genes also feed into many significantly upregulated biological processes, including cholesterol and steroid metabolism (Figure 2). The increase in activities and expression of metabolic pathways and enzymes seen in this manuscript and by other researchers may be a response mechanism by which the cell senses abnormal steroid levels and seeks to develop the appropriate precursor chemicals that can be used to offset the unnatural steroid ratios.

One of the potential uses of –omics data is the investigation of novel biomarkers to evaluate chemical exposures on wildlife [13,14]. One of the most widely used biomarkers of androgen exposure is *spiggin*, a glycoprotein synthesized in the kidneys of male three-spined stickleback (*Gasterosteus aculeatus*) via androgen signaling [23]. In female *G*. *aculeatus*, this protein was found to be upregulated in the kidneys after exposure to 1 μg/L of methyltestosterone (MT), 5 μg/L of dihydrotestosterone (DHT), and 5 μg/L of TB, with co-treatment of the anti-androgen flutamide (flu) resulting in decreased *spiggin* protein levels [23,24]. These results indicate that this gene may be a direct target of androgen action and that *spiggin* has a strong potential to be developed as a robust biomarker for androgen exposure in *G*. *aculeatus*. However, this species’ restricted distribution to colder waters and the inability to use this protein in species outside of the stickleback family (*Gasterosteidae*) imposes limitations for this androgen biomarker’s widespread usage.

Expression of the nuclear receptors for androgen ligands, such as *androgen receptor β* (*ARβ*), is another potential biomarker of androgen exposure [25]. In *G*. *aculeatus*, *ARβ* mRNA was expressed in liver tissues at a detectable yet significantly lower level than in the gonads and kidneys, with no significant effects of gender on tissue expression level [26]. *ARβ* is also expressed at comparable levels in adult female and male *G*. *affinis* fin tissues [5] as well as in androgen-treated anal fins of *G*. *affinis* fry [4]. In this manuscript, we found a decrease in *ARβ* in the liver, which may be attributed to a negative feedback of the AR-mediated pathway during subchronic androgen exposure. This would indicate that *ARβ* may not be a suitable candidate as an androgen biomarker for use in a field setting where animals have likely been subchronically exposed. Previous studies in mosquitofish have evaluated the expression patterns of genes expressed in the anal fin tissue, including *shh*, *msxC*, and *fgfr1*[4,12], but the analysis of genes expressed in the liver are of interest for evaluating androgen biomarkers that can be developed in species other than mosquitofish.

Using qPCR, we were able to statistically validate the expression of the genes *17βhsd3* and *zp2*. The *17βhsd3* subtype is known to catalyze the reduction of the androgen precursor androstenedione (ADE) to testosterone (T) [27]. This subtype is predominantly expressed in the testes [28] but is also found at detectable levels in the livers of both male and female zebrafish, albeit at higher levels in males [27]. Members of this enzyme family are also upregulated in mice adipose tissue after exposure to DHT [29]. We hypothesize that upregulated expression of this enzyme is a feedback mechanism of the hypothalamus pituitary gonadal (HPG) axis in response to changes in levels of sex steroids. Changes in sex steroid levels were seen during androgen exposures including a decrease in both T and E2 during exposures of female fathead minnows to 0.5 and 5 μg/L of TB [30]. Because of its expression in other species and its relationship to the endocrine system, further experimentation on the utility of this gene as a biomarker of subchronic androgen exposure by field work validation is warranted.

During oocyte maturation, zona pellucida (ZP) glycoproteins are synthesized in the liver and deposited into the blood, where they are subsequently taken up by gonads [31]. *Zp2* was found to be downregulated in the gonads of female fathead minnow exposed to 1 μg TB/L for 4 days [19], and this gene was also decreased in this study. In addition to *zp2*, the subtype *zp3a*.*1* was also found to be downregulated by TB exposure in this microarray data set (Additional file 1). Also found in female fathead minnows exposed to 1 μg TB/L for 4 days were several forms of the egg yolk precursor protein *vtg*, which were downregulated by TB exposure, including *vtg6*, *vtg2*, and *vtg1*[19]. In the previously published study on *vtg* gene expression in these samples, we found a significant downregulation in *vtg1* by qPCR [12] and several *vtg* subtypes were also significantly downregulated in this microarray data set (Additional file 1). This decrease is hypothesized to be a result of the indirect anti-estrogenic effects of TB exposure through endpoints such as a decrease in E2 levels, which lead to decreased levels of estrogen-regulated genes such as *vtg*[30]. In addition, a dysregulation of steroid metabolic enzymes during androgen exposure may also influence the normal activities of the HPG axis [32]. Future studies evaluating *zp2* during androgen exposure can provide additional insights into the reproductive impacts of androgenic chemicals on the HPG axis of aquatic organisms and can complement data on *vtg* in both a laboratory and field setting.

## Conclusions

We developed a custom 8 × 15,000 gene microarray for *G*. *holbrooki* and utilized this microarray to evaluate changes in global hepatic gene expression after exposure to a potent androgen receptor agonist. Over 500 genes were significant (p < 0.05, FDR α = 0.05) which had greater than a 1.5 fold change from the controls. Microarray data generated from this study provide insights into the mechanisms of endocrine disruption in this species, most notably the significant upregulation of the biological processes involved in cholesterol and lipid metabolism after subchronic androgen exposure. The similarities in the metabolic pathways being modified during exposure to TB and to other androgenic chemicals provide insights into the biological impacts of androgen exposure. We have validated an increase in the expression of the steroid metabolism gene *17βhsd3* and will validate this gene’s expression in future research at paper mill impacted field sites that are believed to be contaminated with androgenic compounds. In addition, we saw a decrease in the expression of numerous subtypes of key genes involved in oocyte development and function such as *vtg* and *zp*, suggesting that androgen exposure can negatively impact the reproductive abilities of this species.

The *G*. *holbrooki* microarray will allow for this non-model species to become more fully developed into a bioindicator organism for endocrine disrupting pollutants and future studies will enhance knowledge of the biology and toxicological response of this species. While microarray data generated from this and other studies do not provide a full understanding of the molecular mechanism of chemical exposure, it provides a foundation of knowledge and tools that can be used to delve further into understanding the biology of *G*. *holbrooki* and how this organism can be used as a robust bioindicator organism for endocrine disrupting pollutants in the environment.

## Methods

### cDNA library construction

Tissues from livers, gonads, and brains of multiple male and female mosquitofish, anal fin tissues from males, and whole-body < 2 week-old fry were used as starting material for cDNA library construction. RNA was isolated from these tissues using TRIzol reagent (Invitrogen, Grand Island, USA). In brief, tissues were homogenized in TRIzol (1 mL), incubated at room temperature for 5 minutes, and centrifuged at 12,000 x G for 15 minutes. A 1/5 volume of chloroform (200 μL) was added and after a 10 minute incubation the organic and aqueous layers were separated centrifugation at 12,000 x G for 10 minutes. Isopropanol was used to precipitate the RNA out of the aqueous layers and the RNA pellet was washed twice with ethanol. All samples were rehydrated using RNAsecure reagent (Ambion, Grand Island, USA). The quality and quantity of the RNA was determined using the Nanodrop (ThermoScientific, Waltham, USA) as well as the 2100 BioAnalyzer (Agilent, Santa Clara, USA). The range of A_260_/A_280_ values was 1.96 to 2.20 and the RNA integrity number (RIN) range was 7.7-9.9. Samples were not DNase-treated per the manufacturer’s recommendation for cDNA library construction.

Equal masses of RNA per tissue type and sex were pooled into a single 3 μL RNA sample of 500 ng/μL. cDNA library construction using the MINT-Universal kit (Evrogen, Moscow, Russia) was conducted per the manufacturer’s recommended protocol. In brief, first strand cDNA synthesis adapter ligation was conducted for 2 minutes at 70°C and immediately followed by reverse transcription for 2 hours at 42°C. Evaluative PCR was conducted to determine the optimal number of cycles to utilize for double-stranded cDNA synthesis and 8 μL of the PCR reaction was analysed on a 1.2% agarose gel. Based on the agarose gels of all cycle numbers tested and following the manufacturer’s recommendation of avoiding both under and over-amplification of the cDNA library, 21 PCR cycles was selected. Full-size double-stranded cDNA was amplified using the following PCR protocol: 95°C for 1 min; 21 cycles of 95°C for 15 s, 66°C for 20 s, and 72°C for 3 min; 66°C for 15 s; 72°C for 3 min. The cDNA library was purified using the Wizard SV Gel and PCR Clean-Up System (Promega, Fitchburg, USA).

To reduce the presence of overly abundant transcripts, the TRIMMER kit (Evrogen, Moscow, Russia) was utilized. In brief, 1200 ng of the purified cDNA library was incubated with hybridization buffer for 5 hours at 68°C. A 1X solution of the double-stranded nuclease (DSN) enzyme was determined to be the appropriate concentration of enzyme to achieve a balance between under and over-digestion of transcripts as per the manufacturer’s protocol recommendations. DSN was added to the solution for 25 minutes at 68°C and the reaction was stopped by DSN Stop Solution incubation for 5 minutes at 68°C. First amplification of normalized cDNA was PCR-amplified using the following protocol: 95°C for 1 min; 22 cycles of 95°C for 15 s, 66°C for 20 s, and 72°C for 3 min. The cDNA library was purified using the Wizard SV Gel and PCR Clean-Up System (Promega, Fitchburg, USA).

### Sequencing and gene annotation

The normalized cDNA library was first sequenced using the Genome Analyzer IIx (Illumina, San Diego, USA) on a single lane at the University of Florida’s sequencing core facility. A second round of sequencing on a ¼ plate of the 454 XLR70 GS-FLX (Roche, Basel, Switzerland) was later conducted, also at the University of Florida’s sequencing core facility.

An initial assembly of data generated from the Illumina GAIIx was performed using Assembly by Short Sequences (ABySS) version 1.2.6 [33]. The programs of cross-match in Phrap and cleanup module in Paracel Transcript Assembler (PTA) version 3.0.0 (Paracel Inc, Pasadena, CA) were used for assembling raw Illumina reads and to remove low-quality reads. Low-quality end regions were trimmed and resulting reads of ≥ 30 bp were kept for subsequent assembly. The assembled contigs of the Illumina run of ≥ 400 bp were kept for further analysis.

Co-assembly of the 454 reads, as well as the assembled ≥ 400 bp contigs and cleaned reads generated from the Illumina data (see above), was performed with the Newbler Assembler (Roche, Basel, Switzerland). A final assembly of all data sets (i.e. the co-assembled 454 reads, the assembled ≥ 400 bp Illumina contigs, and cleaned reads from the Illumina run) by PTA was also conducted, with the addition of previously unassembled 454 reads. All sequences were checked for universal and species-specific vector sequences, adaptors, and PCR primers used to create cDNA libraries. *Escherichia coli* contamination and mitochondrial and ribosomal RNA genes of *Catostomidae* (contaminants potentially introduced during the dissection process) were identified and removed from input sequences, and poly A/T tails and intrinsic repeats that were identified in *Homo sapiens* were annotated prior to clustering and assembly. Low base-call quality data were trimmed from the ends of individual sequences and the sequences with length < 75 bp were excluded from consideration during initial pair-wise comparison. After clean-up, sequences were passed to the PTA clustering module for pair-wise comparison and then to contig assembly program 3 (CAP3)-based PTA assembly module for assembly.

Large-scale homology searches of the resulting sequences against the NCBI NR and NT databases were conducted using an in-house computation Basic Local Alignment Search Tool (BLAST) pipeline. To obtain a more complete description of gene function for each query sequence [34], the top 100 BLAST hits were retrieved and the best scoring BLAST hit and tentative GO classification with e-value ≤ 1^e-5^ were annotated to query sequences. GO term assignments were organized around GO hierarchies and divided into biological processes, cellular components, and molecular functions. The assembled sequences were also characterized with respect to functionally annotated genes by BLAST searching against the NCBI reference sequences (RefSeq) of the model organisms *Homo sapiens* (38,556 sequences) and *Danio rerio* (31,154 sequences). Queries were considered to have a clear homolog of the search organism when the results had an e- value ≤ 1^e-5^, the length of the aligned segment was ≥ 50 bp, and the identity was > 85%.

To find a maximum set of non-redundant sequences representing unique genes for probe selection, genes were selected from the BLAST results using the e-values ≤ 1^e-7^ and ≤ 1^e-10^ as the cut-off thresholds in the NR & NT search and the RefSeq search respectively. The selected sequences were then submitted to Agilent for probe design. Several genes that were not found in the cDNA library sequences but had been previously cloned from *G*. *holbrooki* were also included (*shh*, *ARβ*, *rack1*, *fgfr1*, and *msxC*). This microarray design has been deposited onto the Gene Expression Omnibus (GEO) database (GPL16784). Custom microarrays were printed in an 8 X 15,000 format (Agilent, Santa Clara, USA).

### Samples used for microarray analysis

Four liver tissues from a 14-day exposure of female *G*. *holbrooki* to 1 μg/L of the potent androgen receptor agonist 17β-trenbolone (17β-hydroxyestra-4,9,11-trien-3-one; abbreviated as TB) and four liver tissues of female *G*. *holbrooki* exposed to the vehicle control (ethanol) [12] were used to determine genes that were significantly differentially regulated during a subchronic androgen agonist exposure. This time point and dose of TB were selected for microarray analysis as they represent the first time point of significant anal fin elongation at this dose, as well as a dose and time point in which mRNA expression of *vitellogenin* was significantly downregulated [12]. RNA was isolated from the livers as previously described using TRIzol (Invitrogen, Grand Island, USA), rehydrated using RNAsecure (Ambion, Grand Island, USA), and DNase treated using the Turbo DNA-free kit (Ambion, Grand Island, USA). All oocyte-development stage-matched RNA samples per treatment were evaluated for RNA integrity using the 2100 BioAnalyzer (Agilent, Santa Clara, USA). The range of RIN values was 8.3-8.9.

### Microarray labeling and hybridization

For amplification and labeling, the Quick Amp Labeling kit (Agilent, Santa Clara, USA) was utilised with adjustments made for a half reaction, one color (Cy3) protocol. In brief, T7 primers were annealed to 1000 ng RNA with the addition of RNA spike-in controls (One color RNA spike-in mix; Agilent, Santa Clara, USA) by incubation at 65°C for 10 minutes. cDNA synthesis using MMLV-RT was conducted at 40°C for 2 hours followed by 65°C for 15 minutes. cDNA was reverse-transcribed into cRNA using a T7 RNA polymerase with the addition of Cy3 to the reaction. *In vitro* transcription proceeded at 40°C for 2 hours. Cy3-labelled cRNA was purified using the RNeasy kit (QIAGEN, Hilden, Germany) and eluted in nuclease-free water. The cRNA concentration and specific activity (pmol Cy3 per μg cRNA) was determined using the Nanodrop (ThermoScientific, Waltham, USA); only samples with a specific activity > 8 were used for downstream procedures.

For each sample, 600 ng of purified Cy3-labeled cRNA was hybridized to the custom *G*. *holbrooki* microarray using the Gene Expression hybridization kit (Agilent, Santa Clara, USA). Cy3-labeled cRNA was incubated with blocking agent and fragmentation buffer at 60°C for 30 minutes. Hybridization buffer was then added to the sample and 40 μL of the sample was added to the gasket. The slide and gasket were incubated for 17 hours at 65°C while rotating at 10 rpm. The slide was then washed, dried, and scanned at the University of Florida gene expression core using the Microarray Scanner (Agilent, Santa Clara, USA). All microarray data were deposited into the GEO database (GSE45261).

### Microarray data analysis

Each sample was evaluated for quality controls before downstream data processing and analysis. These include a lack of blank spots on the scanned image and the presence of clear green spots in the 4 corners of each array sample. The distributions of each of the signal plots were normal with < 1% of non-uniform features. The R^2^ value of the spike-in curves were all > 0.95 and all other evaluation metrics were within normal range. All subsequent data analysis was conducted on background subtracted signals with non-uniform spots flagged and discarded for subsequent analyses. Microarray data were analyzed using JMP Genomics 6.0 (SAS Institute, Cary, USA) and all data were log2 transformed. Parallel plots and box plots were used to evaluate the uniformity and distribution of the signals respectively. Data were loess-normalized to achieve a uniform distribution of signals.

Differences in mean gene expression values between the control and TB-exposed female *G*. *holbrooki* hepatic gene expression profiles were determined by a one-way ANOVA, with p < 0.05 as the significance level and a FDR of α = 0.05 to evaluate errors by multiple testing. For subsequent analysis, genes with greater than 1.5 fold increases or less than −1.5 fold decreases versus the controls and p < 0.05 were utilized. Hierarchical cluster analysis was performed on these data with Cluster 3.0 using median-centered expression data by centered correlation and complete linkage clustering [35]. The resulting expression map was visualized using Java Treeview 1.1.6r2 [36].

Annotation analysis was used to find enriched and under-represented GO Biological Processes by Fisher’s exact test using JMP Genomics 6.0 (SAS Institute, Cary, USA). The resulting Fisher raw p-value was used to determine enriched or under-represented GO Biological Processes with a significance level set at p < 0.05 and a FDR of α = 0.05. This analysis determines the Biological Processes with a greater (enriched) or lesser (under-represented) number of significantly differentially regulated genes observed than is expected in the category by chance. PathwayStudio™ (Elsevier, Amsterdam, The Netherlands) was used to visualize pathways and genes linked to significantly enriched or under-represented biological processes based on ResNet 9.0 database of human homologues.

### Follow-up qualitative polymerase chain reaction analysis

Five genes were selected for expression validation by qPCR. Primers were designed using the Primer3 software [37] based on the *G*. *holbrooki* gene sequence derived from the cDNA library sequence data; see Additional file 2 for primer sequences and GenBank ascension numbers. Three of these genes (*17βhsd3*, *ARβ*, and *zp2*) were cloned into the pGEM-T-easy vector (Promega, Fitchburg, USA) and a serially-diluted standard curve was analysed to determine PCR efficiency. The efficiencies of the primers for two of the genes (*ATF1* and *acaa2*) were determined using serial dilutions of RNA. *Rpl8* was used as an internal standard; this gene has been previously demonstrated to have consistent expression in the samples used for microarray analysis [12]. The average qPCR efficiency and slopes of all genes analysed was 98.21% and −3.379 respectively.

All eight of the *G*. *holbrooki* liver samples exposed to the vehicle control or 1 μg TB/L for 14 days [12] were used for follow-up qPCR analysis. 2 μg of DNase-treated RNA was used for reverse transcription by M-MLV (Promega, Fitchburg, USA). Real-time qPCR analysis was conducted using the myiQ single color real-time PCR detection system (BioRad, Hercules, USA). In brief, 200 ng of cDNA was PCR-amplified using SYBR Green Supermix (BioRad, Hercules, USA) in a 96-well plate format. Quality controls were analysed on each plate (a negative template control of water only and a negative RT control) and all samples and standards were run in duplicate. A two-step protocol with the following parameters was utilized: 95°C for 3 min, 40 cycles of 95°C for 10 sec followed by a 1 min annealing step at 58°C (except for *rpl8* at 60°C), followed by a melt curve with 30 sec intervals at 0.5°C temperature increases (range 55–95°C). Amplification data were analyzed with the iQ5 software (BioRad, Hercules, USA) and statistical analysis was conducted using the delta delta Ct method. For this analysis, the average *rpl8* Ct value was subtracted from the average gene Ct value and standard deviation was calculated as the square root of the sum of the gene’s standard deviation squared and *rpl8*’*s* standard deviation squared. Fold changes were calculated as 2^^(−delta delta Ct value)^. Statistical significance was evaluated using the Student’s T-test with a p < 0.05 set as the significance threshold. Melt curves of all samples contained a single peak, indicating that no secondary products or primer dimers formed during the reaction. There was an average difference between the sample with the lowest Ct and the NTC of 6.01 cycles and an average difference between the minus RT and its corresponding reverse transcribed sample of 8.74 cycles. The variability of the *rpl8* signal was 4.06%.

## Abbreviations

17βhsd3: 17β-hydroxysteroid dehydrogenase; ABySS: Assembly by Short Sequences; acaa2: Acetyl-CoA acyltransferase 2; ADE: Androstenedione; ANOVA: Analysis of variance; apoA-1: Apolipoprotein A1; ARβ: Androgen receptor β; ATF1: Activating transcription factor 1; BLAST: Basic Local Alignment Search Tool; CAP3: Contig assembly program 3; DHT: Dihydrotestosterone; DSN: Double-stranded nuclease; E2: Estradiol; EDC: Endocrine disrupting chemical; ERE: Estrogen response element; FDR: False discovery rate; fgfr1: Fibroblast growth factor receptor 1; GO: Gene ontology; HPG: Hypothalamus pituitary gonadal axis; rpl8: Ribosomal protein L8; msxC: Muscle segment homeobox C; MT: Methyltestosterone; PTA: Parcel transcript assembly; qPCR: Quantitative polymerase chain reaction; RIN: RNA integrity number; T: Testosterone; TB: 17β-Trenbolone; Shh: Sonic hedgehog; Vtg: Vitellogenin; zp2: Zona pellucida 2.

## Competing interests

The authors declare that they have no competing interests.

## Authors’ contributions

EB isolated RNA and developed the *G*. *holbrooki* cDNA library using the MINT cDNA synthesis and TRIMMER kits, isolated RNA from the tissue samples used for the microarray analysis, conducted the cRNA amplification and microarray hybridization, analyzed the microarray data, and conducted the qPCR analysis. FY completed the sequence assembly, annotation, and helped design the *G*. *holbrooki* microarray. DM prepared cDNA libraries from *G*. *holbrooki* for sequencing by Illumina and 454. TB provided samples for the cDNA library construction and conceived the idea for the construction of the custom microarray. ND led the experimental design and framework development for the construction of the microarray. All authors read and approved the final manuscript.

## Supplementary Material

Additional file 1 **List of all significant transcripts.** Spread sheet containing annotation information and fold change levels of all transcripts that were significantly differentially regulated as determined by a one-way ANOVA (p < 0.05). Click here for file

Additional file 2 **GenBank accession numbers and primers utilized in this study.** Table with list of GenBank accession numbers and primer sequences used for cloning and qPCR. Click here for file
