# Supplementary material for: Custom microarray construction and analysis for determining potential biomarkers of subchronic androgen exposure in the Eastern Mosquitofish (Gambusia holbrooki)
Source: BMC Genomics. 2013 Sep 28;14:660. doi: 10.1186/1471-2164-14-660 (PMC3852779; doi:10.1186/1471-2164-14-660)
Supplement: Additional file 2 — GenBank accession numbers and primers utilized in this study. Table with list of GenBank accession numbers and primer sequences used for cloning and qPCR. [file 1471-2164-14-660-S2.docx]

| **Additional File 2.** GenBank accession numbers and primers utilized in this study | | |
| --- | --- | --- |
| Gene name | GenBank accession # | qPCR primers (5’ to 3’) |
| *17-beta hydroxysteroid dehydrogenase 3* | KC763475 | Forward: GCAAGGCTTACAGGACTACGTT  Reverse: TCACAGCATCCATCGGTTC |
| *Activating transcription factor 1* |  | Forward: GCCCTGAAAGACATCTACGG  Reverse: TGTCAGCGCGTTTGTTATTC |
| *Acetyl-CoA acyltransferase 2* | KC763476 | Forward: GAGCATTATGGGGATTGGTC  Reverse: CCTTGGCAACGGAAAGATAC |
| *Androgen receptor beta* | AB182329.1 | Forward: AGAACGTGCCTGATTTGCTC  Reverse: ATGGTGCAGTCGTTTTTGCT |
| *zona pellucida glycoprotein 2* | KC763477 | Forward: CAGTGGGTCCTTCATCTGGT  Reverse: GGGTTCCATTGAATTGGTGT |
| *Ribosomal protein L8* | DQ865277 | Forward: AACTACGCCACCGTCATCTC  Reverse: CAGGATGGGCTTGTCGATAC |
